# Paleotemperature, geochemical and grain size data in Quaternary sediments from the Gloria Drift (Northwest Atlantic)

**DOI:** 10.1016/j.dib.2018.05.105

**Published:** 2018-05-23

**Authors:** Leyla Bashirova, Evgenia Dorokhova, Vadim Sivkov, Ekaterina Novichkova

**Affiliations:** a Shirshov Institute of Oceanology, Russian Academy of Sciences, 36, Nahimovskiy prospekt, Moscow, Russia; b Immanuel Kant Baltic Federal University, Nevskogo street, 14, 236041 Kaliningrad, Kaliningrad region, Russia

**Keywords:** Sea surface temperature, Ice-rafted debris, Stable isotope data, Sediment grain size, Quaternary sediments, North Atlantic, Gloria Drift

## Abstract

Data file presents information on the variation in sea surface temperatures (SST), as well as geochemical (e.g. stable isotope, calcium carbonate), micropaleontological and grain size data from the Gloria Drift (Northwest Atlantic). The data are obtained from the three marine sediment gravity core sections (AMK-4493, AI-3646 and AI-3415) which were formed during Quaternary period. Dataset contains SST values (winter and summer: 0–50 water layer) and ice-rafted debris (IRD) counts, each in 308 samples; stable isotope data (δ^18^O and δ^13^C) from 235 samples; calcium carbonate content from 351 samples; relative abundance of polar species of planktonic foraminifera *Neogloboquadrina pachyderma* (sinistral) (Ehrenberg) in 51 samples, data of grain size analysis in bulk sediments (123 samples) and carbonate-free sediments (664). These data provide information about conditions of sedimentation at the Gloria Drift area.

**Specifications Table**

**Table:** 

Subject area	*Marine Geology*
More specific subject area	*Sedimentation in the deep ocean basins*
Type of data	*1 Table*
How data was acquired	*Microscope, laser analyzer, carbon analyzer, mass spectrometry*
Data format	*Raw*
Experimental factors	*Marine sediment samples were washed with distilled water and sieved to receive fraction >150* *μm for micropaleontological and IRD counts.*
	*For grain size analyze of carbonate-free sediments: organic matter and carbonates were removed from sediments by treatment with excess H*_*2*_*O*_*2*_*and HCl, respectively. To disaggregate component grains, sodium tripolyphosphate was added and then each sample was sonicated with an ultrasonic bath immediately before analysis.*
	*Other data were obtained using a standard laboratory treatment.*
Experimental features	*At least 300 lithic grains (IRD) and foraminiferal shells were counted per one sample.*
	*At least 20–30 foraminiferal shells of N. pachyderma (s) were used for stable isotopes measurements.*
	*SST were reconstructed using Modern Analog Technique [MAT; Prell].*
	*Other data were processed and analyzed using a standard laboratory treatment.*
Data source location	*Shirshov Institute of Oceanology*, Russian Academy of Sciences*, Moscow, Russia*
Data accessibility	*Data are presented with this article*

**Value of the data**

• Data on SST, IRD, calcium carbonate content, ratio of *N. pachyderma* (s), as well as stable isotope data from the marine sediments provide an information about past climate conditions in the studied area during the Quaternary.
• Grain size data allow to reconstruct conditions of sedimentation, as well as near-bottom currents intensity in the past.
• Main research output from use our dataset is reconstruction of sedimentation processes at the Gloria Drift area during Quaternary period.

## 1 Data

Data file contains quantitative information from the three sediment cores recovered from the Gloria Drift: SST, IRD, stable isotope data (δ^18^O and δ^13^C), calcium carbonate content, relative abundance of polar species of planktonic foraminifera *N. pachyderma* (s) in AI-3646 core, grain size data of bulk and carbonate-free sediments. Data provide information on the changes in conditions of sedimentation at the Gloria Drift area during the Quaternary period.

## 2 Experimental design, materials, and methods

**Fig. 1:**
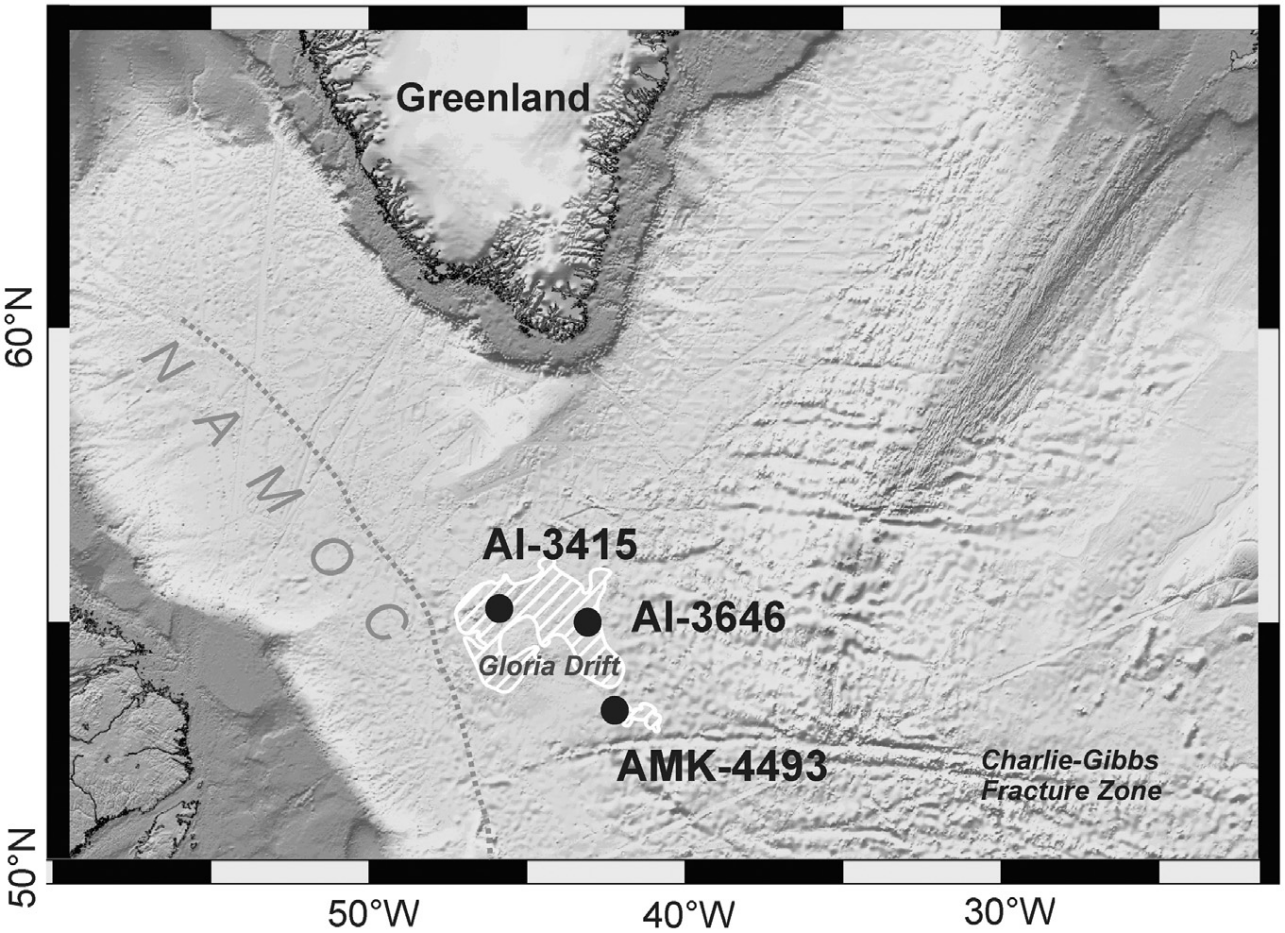
Location of the sediment cores which are provided with data files. NAMOC – Northwest Atlantic Mid-Ocean Channel.

All data are obtained from three marine sediment cores recovered from the Gloria Drift (Fig. 1; Table 1). Data were collected during the 48th cruise of the R/V “Akademik Mstislav Keldysh” (2002; AMK-4493 core), as well as during 49^th^ (2015; AI-3415 core) and 51st (2016; AI-3646 core) cruises of R/V “Akademik Ioffe”.

**Table 1:** Location of sediment cores.

Sediment core	Latitude, N	Longitude, W	Water Depth, m	Core length, m	Area
AMK-4493	53°31.22’	42°45.74’	3547	3.69	Southeastern slope of the Gloria Drift
AI-3646	55°0.124’	43°45.393’	3346	5.05	Northeastern slope of the Gloria Drift
AI-3415	55°34.313’	46°12.559’	2985	5.07	Northwestern slope of the Gloria Drift

For micropaleontological analysis and IRD counts sediments were washed with distilled water and sieved to receive fraction >150 μm. IRD data and planktonic foraminiferal shells were counted under an MBS-10 microscope. At least 300 lithic grains (IRD) or foraminiferal shells were counted per one sample. IRD index is expressed as a number of lithic grains per gram of dry sediment. Relative abundance of planktonic foraminifera in AI-3646 core sediments is presented. SSTs were reconstructed using a Modern Analog Technique (MAT; water layer 0–50 m) [1]. Modern faunal [2] and hydrological [3] databases were applied.

The δ^18^О and δ^13^С fluctuations in the planktonic foraminiferal shells (*N. pachyderma* (s), ≥30 specimens, average size ~150 μm) were analyzed at the Leibniz Laboratory (Kiel University) using a Finnigan MAT 253 mass spectrometer connected to a Kiel IV carbonate preparation device. The analytical precision for analyzing stable carbon and oxygen isotopes is <0.05‰ and <0.08‰, respectively. The CaCO_3_ content was determined using a coulometric method with an AN-7529 M express analyzer.

Grain size analysis was carried out on the SALD-2300 Laser Diffraction Particle Size Analyzer (Shimadzu, Japan). Organic matter and carbonates were removed by treatment with excess H_2_O_2_ and HCl, respectively. At some horizons, grain size analysis was carried out without chemical pretreatment (bulk sediments). To disaggregate component grains, sodium tripolyphosphate was added and then each sample was sonicated with an ultrasonic bath immediately before analysis.

To determine the boundaries between marine isotope stages we used stable isotope, micropaleontological, carbonate content, and IRD data.
